# New Insights on the Neglected Avian Nematode *Hystrichis tricolor*: Hystrichiosis-Induced Proventriculitis in Synanthropic Egyptian Geese (*Alopochen aegyptiaca* Linnaeus, 1766) in Germany

**DOI:** 10.3390/pathogens12050663

**Published:** 2023-04-29

**Authors:** Ella F. Fischer, Elfi K. Schlohsarczyk, Manuela Gröf, Ulrich Gärtner, Anja Taubert, Carlos Hermosilla

**Affiliations:** 1 Biomedical Research Centre Seltersberg (BFS), Institute of Parasitology, Justus Liebig University Giessen, Schubertstr. 81, 35392 Giessen, Germany; anja.taubert@vetmed.uni-giessen.de (A.T.); carlos.r.hermosilla@vetmed.uni-giessen.de (C.H.); 2 Institute of Veterinary Pathology, Justus Liebig University Giessen, 35392 Giessen, Germanymanuela.groef@vetmed.uni-giessen.de (M.G.); 3 Institute of Anatomy and Cellular Biology, Justus Liebig University Giessen, 35392 Giessen, Germany

**Keywords:** *Hystrichis tricolor*, *Alopochen aegyptiaca*, endoparasites, Egyptian goose, proventriculus

## Abstract

*Hystrichis tricolor* is a neglected avian enoplid nematode (superfamiliy Dioctophymatoidea) and known to parasitize various species of the Anatidae (*Anas* spp. and *Mergus* spp.) from the northern hemisphere, inducing mainly proventriculitis in domestic and wild waterfowl. Here, we focus on the pathological findings of naturally *H. tricholor*-infected Egyptian geese (*Alopochen aegyptiaca*) and a neozoan shelduck (Tandorninae) of Germany. Nowadays, this species is considered the fastest-spreading alien waterfowl species within Western Europe. Additionally, molecular sequencing coupled with phylogenetic characterization of *H. tricolor* is reported. *Post mortem* analyses unveiled patent gastric *H. tricolor* infections in eight of twelve infected birds (8/12; 66.7%), inducing proventriculitis resulting in large visible nodular lesions. Histopathological findings point to chronic host pro-inflammatory immune reactions. These results demonstrate the potential role of Egyptian geese as natural reservoir hosts of *H. tricholor* and highlight their possible role in parasite transmission (i.e., spillback) into endemic waterfowl species. Due to avian health concerns, the occurrence of hystrichiosis should be monitored in native waterfowl in the future, introducing appropriate management practices in conservation strategies of endemic wild birds not only in Germany but elsewhere in Europe.

## 1. Introduction

*Hystrichis tricolor* (Dujardin, 1845) is an enoplid nematode of the genus *Hystrichis*, which currently includes ten species [1], which are *H. coronatus* (Molin, 1861), *H. pachicephalus* (Molin, 1861), *H. corvi* (Hendricks, 1969), *H. acantocephalicus* (Molin, 1861) and *H. africanus* (Vuylsteke, 1964). Four species, (*H. neglectus* (Cram, 1927), *H. orospinosus* (Molin, 1858), *H. wedli* (Cram, 1927) and *H. variospinosus* (Von Linstow, 1879)), have been inadequately described or have been referred as rather immature stages of *H. tricolor* instead of being considered as a separate species [1,2]. Thus, *H. tricolor* has been reported in few publications in the literature as a neglected avian gastrointestinal parasite of different aquatic birds in the northern hemisphere [3,4,5,6]. This avian nematode species belongs to the family Dioctophymatidae, parasitizing diverse domestic and wild waterfowl species (mainly *Anas* spp.), and also including fish-eating waterfowl (mainly *Mergus* spp.) [7,8].

**Figure 1:**
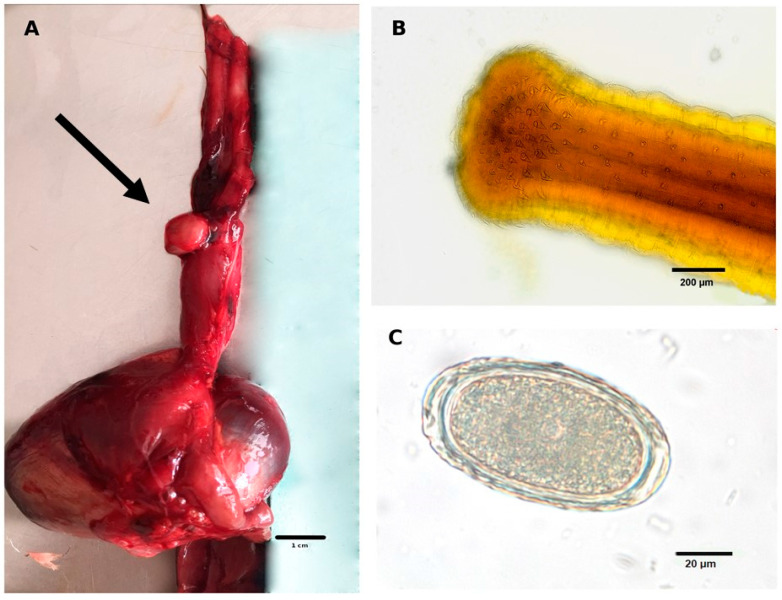
Morphological characteristics of *Hystrichis tricolor* and pathological findings associated with avian hystrichiosis. (**A**) Macroscopic granulomatous lesion (black arrow) on the outside of the proventriculus induced by *Hystrichis tricolor* adults; (**B**) adult *H. tricolor* specimen with typical spines at the cephalic end; (**C**) un-embryonated egg of *H. tricolor* with its characteristic small depressions all over the eggshell detected in an Egyptian goose (*Alopochen aegyptiaca*) scat sample.

Morphological features of *H. tricolor* include a dilated cephalic anterior part carrying approximately 40 rows of small spines which become smaller towards the back of the body [1,2] (Figure 1). According to Schmidt-Rhaesa [1], the cuticle spines are not exclusively for firm attachment of parasitized submucosa but also for inducing injury. The endogenous parasitic stages of *H. tricholor* probably feed on the sore exudate [1]. The pseudocoel of adult nematodes shines red and the cuticle rather white (Figure 1). Unique to *H. tricolor*, in comparison to other species of the genus *Hystrichis*, is the fact that the middle part of the body is dilatated in female individuals [2]. The females are up to 4 cm in length and larger than males. After copula, gravid females excrete oval, thick-shelled and rough un-embryonated eggs with an average size of 85–90 × 35–40 µm [7]. Small round depressions all over the eggshell (Figure 1C) are also mentioned as a morphological characteristic of *H. tricolor* eggs [9].

The lifecycle of *H. tricolor* is obligate heteroxenous containing intermediate hosts (IH) which include various earthworm species (e.g., *Allolobophora dubiosa pontica* (Pop, 1938), *Eiseniella tetraedra* (Savigny, 1826), *Eophila leoni* (Michaelsen, 1891)) or fish-gill leeches where first-stage larvae (L1) moult into second-stage larvae (L2) and finally into infective third-stage larvae (L3). *H. tricolor* infection of waterfowls occurs after oral ingestion of L3-carrying obligate IH [9]. After consumption and digestion of infected IH, L3 are released into the gut lumen, and then actively burrow into either oesophagus submucosa or proventriculus in order to continue development until reaching adult stages. Adults of *H. tricolor* can live up to 30–45 days post infection (p. i.), thereby causing macroscopic visible granuloma-like lesions at the oesophagus/proventriculus submucosa (Figure 1A) [8]. *H. tricholor*-induced nodules open into the lumen of affected organs where eggs are excreted into the faeces.

A pathognomonic macroscopic finding of avian hystrichiosis includes, according to Avery [3], a “hard-calcified tube” around submucosal nematodes in the centre of a circular swelling on the extraluminal side of the proventriculus. Thus, pre-patent, patent and post-patent avian hystrichiosis result in large granuloma formation, most likely due to pro-inflammatory host immune reactions against pre-adult-, adult- as well as egg-stages, leading to stenosis. Consequently, clinical manifestations of avian hystrichiosis include dysphagia and maybe dyspnoea by the induced pressure on the trachea depending on the sizes of the nodules [7,8].

So far, only few studies on avian hystrichiosis in either endemic wild or domestic waterfowl species (i.e., Anatidae) have been conducted [3,4,10]. There are few studies on the occurrence of this parasite species in single individuals or single avian host species. For example, there is a report on the low prevalence of avian hystrichiosis in the common gallinule *Gallinula geleata* (Brisson, 1760) (10%; 6/56) and in the purple gallinule *Porphyrio martinicu*s (Linnaeus, 1766) (2%; 1/52) in Florida, USA [11]. Another study reports an even higher prevalence of *H. tricolor* in long-billed dowitchers (*Limnodromus scolopaceus* (Say, 1823)) in Mexico (58%; 15/26) [12]. Conversely, very little is known on other suitable avian definitive hosts (DH), on the epidemiology, pathogenicity and host immune reactions. Furthermore, there are numerous retrospective controversial discussions in the literature regarding whether previous descriptions of *Hystrichis* species are not more valid as these reports were based on larval morphological identification [8,9]. Unfortunately, whole genome sequences of *H. tricholor* are still missing for clear species identification. The occurrence of *H. tricolor* infections is rarely mentioned in members of the Anserini, but detailed reports of patent *H. tricolor* infections in wild Anserini are missing. Interestingly, most studies of bird species affected by this nematode exclusively detected ducks [5,10,13] and different Tadorninae as DH for enoplid *H. tricolor* [3]. Currently, to the best of our knowledge, there is one report on avian hystrichiosis occurring in Egyptian geese in the United Kingdom recognized, beside genera of Anatidae, as DH [3].

We report in this study naturally occurring *H. tricolor* infections in Egyptian geese (*Alopochen aegyptiaca*), a non-indigenous avian species (syn. alien) in Central Europe, North America and the Arabian Peninsula. Alien species not only have effects on their new environment and its ecology [14], but also on local economies [15]. Consistently, the Egyptian goose, a member of the Anatidae family, is an endemic bird species of the African Sub-Sahara and the Nile valley [16,17,18]. Recent molecular phylogenetic studies have demonstrated that Egyptian geese are most likely related to shelducks and therefore re-placed within the subfamily Tadorninae, which are more related to the genus *Anas* than to the genus *Anser* [19]. Descendants of European Egyptian geese populations were initially introduced during the 17th century in parks of the United Kingdom and the Netherlands. Since then, Egyptian geese have rapidly spread into urban/peri-urban and rural areas, currently breeding widely in many European cities with natural/artificial water habitats [20,21]. Consequently, neozoan Egyptian geese are predisposed to interact in closeness with humans, domestic and wild, indigenous animals. Currently, in Germany it is permitted by law to hunt Egyptian geese to control the increasing population in particular in Western parts of the country. Cities with high densities of Egyptian geese engage hunters to control synanthropic populations all through the year.

Reports in the literature on naturally occurring *H. tricolor* infections in synanthropic Egyptian geese populations are still missing for Germany and elsewhere. The authors of this report aimed to give a detailed histopathological description of the infected tissue of its avian DH and provide morphological data on the adult- as well as egg-stages of the enoplid nematode *H. tricolor*. This study provides the basis for future investigations or monitoring studies of *H. tricholor* infections in endemic bird species by adding novel molecular data.

## 2. Methods

Adult *H. tricholor* specimens were obtained during necropsy from twelve carcasses (*n* = 12) of Egyptian geese, either shot or killed by car accidents in the metropolitan area of Frankfurt (Main, Hesse, Germany). Ten infected carcasses originated from different public swimming lakes in the city of Darmstadt, one infected carcass was shot to prevent bird strikes in the area of the International Airport Frankfurt (Main) and one infected carcass was found, killed by a car accident at the motorway A5 next to Zeppelinheim. All shot birds were from urban habitats and killed to control the increasing population on the basis of the respective legally defined hunting seasons of the Federal State of Hesse, or shot to prevent bird strikes by the airport avifauna management but not for this investigation. The gastrointestinal tract of these twelve birds was examined in necropsy for adult stages of *H. tricolor*. The proventriculi of four Egyptian geese were stored in 10% neutral buffered formalin for 24 h and submitted for histopathological investigation.

### 2.1. Macroscopic and Microscopic Analysis

The number of macroscopic visible or only palpable nodular lesions was counted and their size was measured. Macroscopic visible nematodes were removed carefully under an Olympus BH-2^®^ microscope (Hamburg, Germany) from submucosal tissues, and thereafter suspended in lactophenol in order to decolorize the specimens. After 30 min, nematodes were removed and analysed for morphological and morphometric features using the “Key of nematodes reported in waterfowl” [2] and other descriptions [1,9] (Figure 1B).

#### 2.1.1. Coprological Analysis

The samples of ingesta of these carcasses were assessed by the sodium acetate acetic acid formaldehyde (SAF) technique. A bean-sized sample of ingesta was mixed very well with 10 mL of SAF solution (i.e., sodium acetate, glacial acetic acid, formaldehyde, water). This mixture was thereafter passed through a sterile gauze and centrifuged for three minutes at 600× *g*. After decanting, 8 mL of sodium chloride solution with 3 mL ethyl ether were added and shaken well. Finally, it was again centrifuged (600× *g*, 3 min) and decanted a second time. The sediment was examined by use of a Leica MZ75 microscope (Wetzlar, Germany) equipped with a SC30^®^, Olympus (Hamburg, Germany) digital camera.

#### 2.1.2. Histopathological Analyses

Cross sections of the proventriculi were processed, embedded in paraffin wax, sectioned at 3 µm, stained with hematoxylin and eosin (H&E) and with Periodic acid-Schiff reaction (PAS), according to routine protocols. The sections were investigated under a light microscope (Nikon 80i, Nikon GmbH (Amstelveen, The Netherlands)).

#### 2.1.3. Scanning Electron Microscopy (SEM) Analysis

Two adult *H. tricolor* nematodes, recovered by necropsy from a shot infected *A. aegyptiaca* obtained from Darmstadt, were transferred on 10 mm glass coverslips (Thermo Fischer Scientific, Schwerte, Germany) pre-coated with 0.01% poly-_L_-lysine (Merck, Darmstadt, Germany) for 10 min at room temperature (RT). Thereafter, nematodes were fixed in 2.5% glutaraldehyde (Merck, Darmstadt, Germany), post-fixed in 1% osmium tetroxide (Merck, Darmstadt, Germany) and washed in distilled water before dehydration and critical point drying with CO_2_. Finally, the nematodes were gold labelled by sputtering and viewed on a Philips XL30^®^ scanning electron microscope at the Institute of Anatomy and Cell Biology, Justus Liebig University Giessen, Giessen, Germany.

### 2.2. Molecular Analyses

#### 2.2.1. Sequencing

DNA of four worms, recovered from two shot carcasses of *A. aegyptiaca*, was extracted using the DNAeasy Blood & Tissue kit (Qiagen (Hilden, Germany)) following the manufacturers’ instructions. Following this, a 768 base pair segment of 18S was amplified. Therefore, a PCR was performed, using the primers Sobo18SFWD 5′ TTTGGTTTTCGGATCTGAGG-3′ and Sobo18SREV 5′ GTACAAAGGGCAGGGACGTA-3′. The PCR conditions were the following: 45 µL reaction volume with 5 µL of the isolated template DNA, 5 µL 10x PCR buffer S (Peqlab, VWR, International GmbH, Erlangen, Germany), 1 µL of each primer, 1 µL dNTPs and 1 µL 5U/µL taq Polymerase (Peqlab, VWR, International GmbH, Erlangen, Germany). The PCR protocol started with an initial denaturation for 5 min at 94 °C followed by 35 cycles at 94 °C for 30 s, annealing at 51 °C for 45 s, extension for 90 s at 72 °C with a final extension for five min at 72 °C. The protocol was modified by the authors from a PCR protocol described by Koehler et al. [22].

The PCR product was visualised by a gel electrophoresis on 1.5% agarose gel with Midori Green Advanced^®^ (Nippon Genetics Europe, Düren, Germany) and extracted using the HiYield GelPCR^®^ DNA Extraction kit (Gauting, Germany). Sanger sequencing was performed by LGC genomics, Berlin, Germany. The sequence was added to the NCBI-GenBank with the accession number OQ561211.

#### 2.2.2. Phylogenetic Analyses

To find gene sequences closely related to the isolated OQ561211 sequence, highly matching sequences were gained from BLAST searches via MegAlign Pro^TM^ (DNA STAR) of the NCBI-GenBank database. Members of the Dioctophymatidae and Soboliphymatidae were selected for phylogenetic relationship investigations according to Koehler et al. [22]. All obtained sequences were cut at a length of 761 base pairs. The only other sequence of *H. tricolor* in the NCBI-database is about 500 base pairs shorter than all other consensus sequences. The alignment was conducted using Clustual Omega in the software MegAling Pro^TM^ (DNA STAR, Version: 17.3.0 (58)) [23]. Following Koehler et al. [22], *Trichinella murrelli* (Murrell, 1989) and *Trichinella nativa* (Britov and Boev, 1972) were used as outlines to create a phylogenetic tree, which was constructed by maximum likelihood analysis under 1000 bootstrap replicates [24,25].

## 3. Results

### 3.1. Macroscopic Findings

The twelve *H. tricolor*-infected Egyptian geese carcasses showed nematode-induced nodular, round-shaped swellings in the wall of the proventriculus. All nodules were rough in palpation and opened into the proventriculus lumen, where the hind body of nematodes were visible. Directly around the nematode in the tissue of the provetriculus were calcified cavities in which the parasite was embedded. They were approximately 0.9 mm × 0.4 mm in size and entangled within themselves. The abundance showed a minimum of three and maximum of seven of these nodular lesions, in sizes ranging from 4.0 mm × 4.0 mm × 4.0 mm up to 10.0 mm × 9.0 mm × 9.0 mm. The nodules were detected mainly in the anterior third of the proventriculus and none of them were found in the oesophagus (Figure 1A). All infected animals (*n* = 12) were equal parts male and female and obtained from urban habitats comprising the metropolitan area of Frankfurt (Main) in the Federal State of Hesse, Germany.

### 3.2. Microscopic Findings

#### 3.2.1. Coprological Findings

Eggs of the parasite were found in the intestinal content in eight of twelve *H. tricolor*-positive birds (66.7%, *n* = 12). This proves that Egyptian geese can act as suitable DH for *H. tricholor* in Germany, thereby contributing to the maintenance of its heteroxenous life cycle. There was no correlation between the nematode burden per DH and the level of tissue damage caused by the parasites. All nematode eggs were oval-shaped, rough with the described typical depressions in the eggshell and on average 67.4 (±6) µm × 37.6 (±5) µm (Figure 1C).

#### 3.2.2. Adult Nematodes

The nematodes removed from the submucosa of the glandular stomach were identified on the basis of morphological and morphometric external characteristics as *H. tricolor* [1,2,7]. These include not only the typical head hooks (Figure 1B), the triangular mouth opening and the dilation of the middle part of female body, but also the conspicuous calcified cavity that surrounded these parasites in the submucosa of the glandular stomach. Moreover, the findings of numerous parasite eggs in the ingesta of infected Egyptian geese confirmed the species diagnosis. Adult *H. tricolor* specimens were found in twelve carcasses within nodules of the proventriculi (Figure 1A).

#### 3.2.3. Histopathological Findings

**Figure 2:**
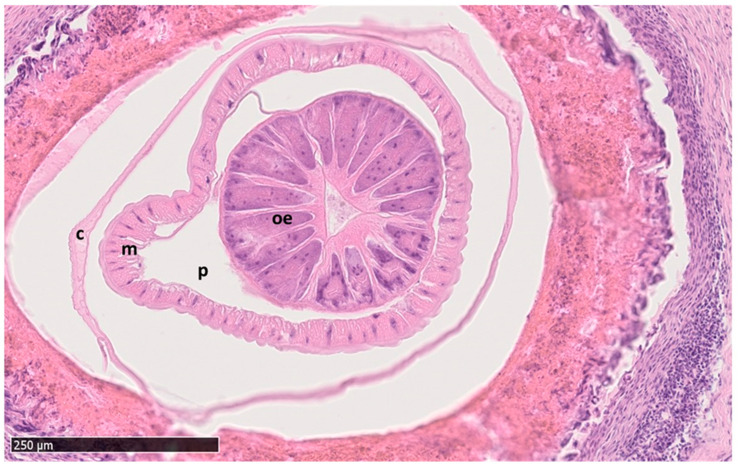
Histological cross section of the nematode *Hystrichis tricolor* surrounded by a granulomatous to pyogranulomatous reaction of the proventriculus tissue. Furthermore, the cuticula (**c**), muscle cells (**m**), body cavity/pseudocoelom (**p**), and the oesophagus (**oe**) with the typical triangular lumen are identifiable. H&E stained.

The submitted proventriculi measured up to 5.5 × 5.0 cm and had a thickness up to 0.7 cm. The *H. tricolor*-infected proventriculi showed a diffusely granular surface of the mucous membrane due to severe follicular hyperplasia, which was accompanied by a moderate amount of lymphocytes, plasma cells and eosinophilic granulocytes. The cross sections revealed multifocal, roughly light beige coloured nodular structures measuring up to 0.4 cm in diameter with a central miliar cavity. They were located within the glandular aspect of the proventriculus bulging into the lumen as well as within the *lamina muscularis* and the serosa. Histologically, these findings were characterized as severe chronic multifocal to transmural granulomatous to pyogranulomatous proventriculitis, accompanied by giant cells (foreign body type) and eosinophilic granulocytes (Figure 2). The centre of the granulomas or pyogranulomas contained amorphous eosinophilic necrotic debris, remnants of necrotic nematodes (400 × 200 µm), oval-shaped, thick-shelled, non-operculated eggs with a coarse surface containing a zygote and which stained positive with the PAS reaction, or longitudinal (2 × 0.5 cm) and cross sections (up to 2.5 × 1.5 mm in diameter) of adult nematodes. The nematodes showed a cylindrical body, a 20 µm thick cuticle with cuticular ridges, a coelomyarian/polymyarian musculature (Figure 2), lateral cords which do not extent significantly farther into the pseudocoelom than the muscle cells, an oesophagus with a triangular lumen and a body cavity (pseudocoelom) containing parts of the male reproductive tract and a digestive tract with an uninucleate cuboidal epithelium. Due to advanced autolytic changes, the intestinal brush border was faintly visible.

#### 3.2.4. Scanning Electron Microscopy (SEM)

**Figure 3:**
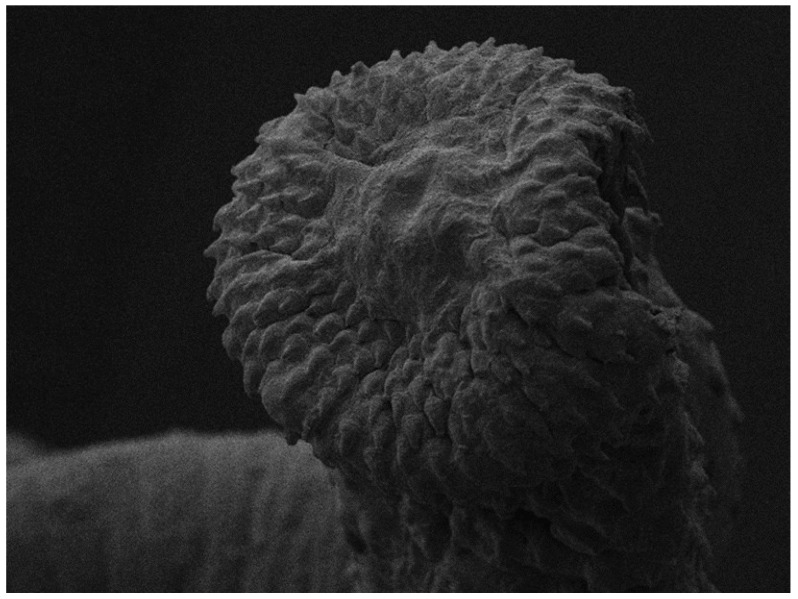
Scanning electron microscope (SEM) image of the anterior end of an adult *Hystrichis tricolor* removed from the proventriculus of an Egyptian goose (*Alopochen aegyptiaca*).

The SEM micrographs confirmed particular species-specific characteristics, such as the dilated anterior end, the slight cone shape and the rows of spines, which decreased towards the middle of the body (Figure 3).

### 3.3. Molecular Findings

#### Sequence and Phylogenetic Findings

**Figure 4:**
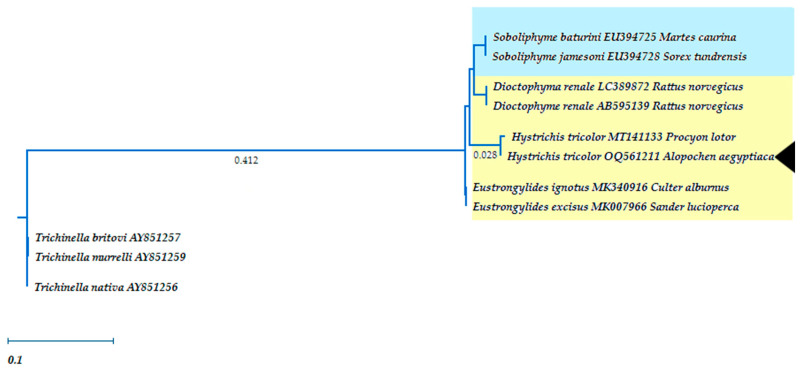
Phylogenetic relationship of *Hystrichis tricolor* to other species of the order Dioctophymatoidea shown in a maximum likelihood tree under 1000 bootstrap replicates estimated from sequences of the 18S gene according to Koehler et al. [22]. Blue: Soboliphymatidae, yellow: Dioctophymatidae including three genera (*Eustrongylides*, *Dioctophyma* and *Hystrichis*).

A 768 base pair region of 18S ribosomal gene was successfully amplified by PCR and sequenced. This allows a closer view of the phylogenetic relationship of *H. tricolor* to other species within the superfamily Dioctophymatoidea (Figure 4). However, it is worth mentioning that there are many species of this family which are not yet sequenced. The phylogenetic analyses suggest that *H. tricolor* varies from other Dioctophymatidae. It differs from the isolates of other genera of this enoplid family. However, its closest phylogenic relation is to *Eustrongylides ignotus* (Jägerskiöld, 1909) (Figure 4). This relation could also be confirmed with two algorithms: the maximum likelihood algorithm as well as the neighbor-joining method. Only one other sequence of *H. tricolor* was placed on the NCBI database [26] and originated from a specimen obtained from a raccoon (*Procyon lotor*) in Italy [27].

## 4. Discussion

This study presents novel insights regarding the biology of *H. tricolor*, describing not only the occurrence of this enoplid nematode in the European Egyptian goose population but also its pathological impact on this alien DH and thereby providing the basis for further investigations on the genus *Hystrichis*.

Regarding pathological findings of avian hystrichiosis, no lesions in the oesophagus were found in all affected Egyptian geese. Therefore, pressure on the trachea through nodules are anatomically not possible. The detected *H. tricolor*-induced nodules could only compress either the lungs, the liver or the heart. However, this is somewhat doubtful based on the birds’ anatomical predispositions. A negative effect of very large nodules on these organs is imaginable in smaller birds acting as DH, such as mallards (*Anas platyrhynchos*), common gallinules or goosanders. Nonetheless, besides potential direct mechanical obstruction of important organs even in larger birds such as the Egyptian goose, the impact of proventricular nodules on whole organism should not be neglected. Wobeser [28] points out that especially a wild bird has a limited amount of energy available, which is used for physiological functions such as reproduction, immunity and growth purposes, which might be hampered in chronic avian hystrichiosis. To cope with pathogenic parasites such as *H. tricolor*, the DH needs sufficient energy supply which might not be available to parasitized individual for its reproduction, growth and/or protective immune reactions [29]. It has been shown in various bird populations that through these adverse scavenging effects, endogenous parasites can significantly influence the host population health [30,31].

The partial sequencing of the 18S gene allows the *H. tricolor* species to be classified within the family Dioctophymatidae. This nematode family includes three genera parasitizing vertebrates: the genus *Dioctophyma*, which includes parasite species of mammalian kidneys, the genus *Eustrongylides* and the genus *Hystrichis*, the latter both burrowing into the oesophagus and/or the proventriculus of aquatic birds [32]. As such, *Diotophyma renale* (Goeze, 1782) is distributed worldwide and known to parasitize the kidneys of different mammals as well as humans. Obligate IH of *D. renale* are various aquatic oligochaete annelids and fishes carrying infective L3 and thereby acting as paratenic hosts (PH) in this life cycle [33]. The closest genetic relation of the species *H. tricolor* consists of the species *Eustrongylides ignotus* (Jägerskiöld, 1909) (Figure 4). The closest relationship to *E. ignotus* was not only confirmed by the maximum likelihood algorithm but also by using the neighbor-joining method. The nematode *E. ignotus* has been reported previously from various members of Ardeidae, Ciconiiformes and Anserini in different parts of the world. Like *H. tricolor* this parasite penetrates the wall of the proventriculus, but the described obligate IH of *E. ignotus* are fishes. However, not all IH are known for this parasite species [34,35]. In the literature, described lesions found in the glandular stomach of *E. ignotus*-infected avian hosts are macroscopically similar to those of avian hystrichiosis. Spalding and Forrester [34] described nodular lesions in the serosal surface of proventriculi of Ciconiformes caused by *E. ignotus-*infections [28,34]. Nevertheless, they also perceived exudates within the air sacks as a consequence of this severe proventriculitis, which limits the ability to breath and consequently compromises the bird’s fitness. Thus, similar pathological findings of eustrongylidosis might be expected to occur for *H. tricolor*-infections. Nonetheless, these findings could not be confirmed in this study. A reason for this might be the way assessed Egyptian geese were culled. In the study on avian eustrongylidosis, all examined birds were euthanized. Conversely, in our study, all birds were shot thereby leading to multiple bleedings into body cavities and/or rupture of inner organs, which clearly impeded our ability to distinguish exudates within the air sacks.

Regarding the epidemiology of avian hystrichiosis, it has been reported to occur with a patchy distribution in certain geographic areas, including hyperendemic-, hypoendemic- and non-endemic foci. For instance, all *H. tricolor*-infected common gallinules were from two lakes at seven sampled sites in Florida (6/56) [11]. Similarly, reports on the epidemiology of avian hystrichiosis in long-billed dowitcher showed that animals allocated in Texas were negative, whereas a very high prevalence was found in animals allocated in Mexico (15/26) [12]. In the current study, all infected animals came from a specific region in Germany, the metropolitan area of Frankfurt (Main), Hesse, and all infected Egyptian geese carcasses showed a high-grade infection of *H. tricolor* and four of them, differing from expectations based on the reports from Avery (1966) [3] and Kavetska (2012) [5], had more than four nematodes in their proventriculi. In studies with more sample sites, many sampled animals and sampling over several years, such as the one published by Kavetska et al. [5], lower prevalence was reported (2/1052). Different plausible explanations for this observation are possible. One explanation of patchy distribution might be linked to the absence of obligate IH in certain geographic areas, thereby not allowing the parasite to fulfil its life cycle; or the pathogenicity of the nematode is so high and restricts the DH so much in its fitness, that it can occur or might be found in only a few DH individuals. Thus, *H. tricholor*-infected individuals may be selected so quickly due to their restricted fitness, or due to the fact that they are rare, as speculated elsewhere [29,31].

The ability of parasites to drive host population dynamics is easily demonstrated in various studies that compared host fecundity of parasite-infected individuals and non-infected ones [36]. Thus, following studies should investigate the clutch size as well as the off-spring success of *H. tricolor*-infected and non-infected Egyptian geese and other native waterfowl species to evaluate the risk for a potential increased spill-back from this alien host.

Taking into account parasite–host interactions, this report provides the first evidence of patent infections of *H. tricolor* in alien Egyptian goose. This finding has epidemiological consequences in the long term and seems essential for further consideration when understanding the biology of this heteroxenous parasite. *H. tricolor* are able to reproduce and pass eggs through Egyptian geese faeces thereby contaminating natural environments where endemic bird species co-habit. The fact that eggs were detected in only 66.7% of all *H. tricolor*-infected birds might be either linked to the pre- and post-patency of avian hystrichiosis, or the lifetime fecundity of individual parasites. Several further factors, described by Rossin et al. [37], are responsible for the amount of excreted eggs, for example, the amount of resources (i.e., nutrients) available for parasite replication, host immunity or the individual size of gravid female nematodes. Taking into account that we did not know at which stage of infection (e.g., pre-patency) the birds were culled no correlation between nodule sizes in proventriculi and nematode burdens were here established. It is also conceivable that the protective host immune system of respective birds might have reacted differently to these parasites.

Another interaction between *H. tricolor* and the host immune system was observed in the size of the eggs excreted by the parasite. The egg size measured here is around 25 µm smaller in length compared to the egg size described by Rommel et al. (85–90 µm × 35–40 µm) [7]. The differences between the nematode eggs detected here and the ones described previously are probably due to the exotic DH. Correlations between the egg size and, for example, host-specific innate immune reactions such as eosinophil concentrations, have already been described for other nematodes [36]. Considerable genetic influences of the host individual on the number and size of eggs excreted by nematodes could also be shown by Stear et al. [38]. In intestinal nematodes, a high variability of egg sizes influenced by the size of the female, has also been described and is not uncommon [29,39].

Overall, our studies underpin the fact that alien Egyptian geese, a member of the Tadorninae, have a closer relationship to Anatinae than to the Anserinae. These alien birds are infected by the neglected enoplid parasite *H. tricolor*, which has a wide host range among waterfowl and has never been detected before in wild Anserinae [8].

## 5. Conclusions

The current study demonstrates the adverse health effects of *H. tricolor*-infections, histologically and macroscopically. A review of other reports on this nematode species shows that comprehensive prevalence studies as well as unambiguous species diagnosis are lacking to assess the impact of this pathogenic parasite on avian host populations. Partial sequencing of the 18S gene of this nematode should be expanded in order to capture the full host range of *H. tricolor*. Our molecularly unambiguous identification of *H. tricolor* provides the basis for future monitoring studies on avian hystrichiosis most probably circulating in endemic birds. Finally, a better understanding of the impact of alien species on the native ecosystem is important in order to prevent native species from extinction. The more we understand the ways in which neozoan invasion occurs, the more we will be able to predict and protect native biodiversity.

## Data Availability

Not applicable.
